# Integrin Alpha8 Beta1 (81): An In-Depth Review of an Overlooked RGD-Binding Receptor

**DOI:** 10.32604/biocell.2025.062325

**Published:** 2025-05-27

**Authors:** Iman Ezzat, Marisa Zallocchi

**Affiliations:** Department of Biomedical Sciences, Creighton University School of Medicine, Omaha, NE 68178, USA

**Keywords:** Transmembrane receptor, cytoskeleton, RGD binding integrin

## Abstract

Integrins are heterodimeric transmembrane receptors that mediate bidirectional interactions between the intracellular cytoskeletal array and the extracellular matrix. These interactions are critical in tissue development and function by regulating gene expression and sustaining tissue architecture. In humans, the integrin family is composed of 18 alpha (α) and 8 beta (β) subunits, constituting 24 distinct αβ combinations. Based on their structure and ligand-binding properties, only a subset of integrins, 8 out of 24, recognizes the arginine-glycine-aspartate (RGD) tripeptide motif in the native ligand. One of the major RGD binding integrins is integrin alpha 8 beta 1 (α8β1), a central Ras homolog gene family member A (RHOA)-dependent modulator highly expressed in cells with contractile function. This review focuses on the recent advances regarding α8β1 function during organ development, with a particular interest in kidney and inner ear development. We also discuss α8β1’s role in injury and disease and its importance for mesenchymal to epithelial transition during cancer development. Finally, we highlight α8β1’s importance for hearing function and its future use as a potential diagnostic and therapeutic tool for disease elimination.

## 1 Introduction

Integrins are a superfamily of cell surface receptors critically involved in mediating interactions between cells and the extracellular matrix (ECM) [1–5]. Since their discovery three decades ago, integrins have been recognized as essential “integrators” linking the extracellular environment to the cytoskeleton and downstream signaling molecules within the cells [2–4]. They are heterodimeric receptors composed of alpha (α) and beta (β) subunits. In humans, the integrin family consists of 18 α and 8 β subunits, forming 24 unique integrin combinations of αβ complexes [6]. Based on their ability to recognize and bind to specific ligands, integrins are categorized into four primary groups: leukocyte cell-adhesion integrins, RGD-binding integrins (which recognize the arginine-glycine-aspartic acid [RGD] motif), collagen-binding integrins (specific to the GFOGER motif), and laminin-binding integrins [7]. The RGD-binding motif is among the most frequently recognized by integrins, with eight family members binding to this sequence: α8β1, αvβ1, αvβ3, αvβ5, αvβ6, αvβ8, α5β1, and αIIbβ3 [8]. This motif is predominantly found in ECM proteins such as fibronectin, nephronectin, osteopontin, vitronectin, and tenascin C [7–9].

**Figure 1:**
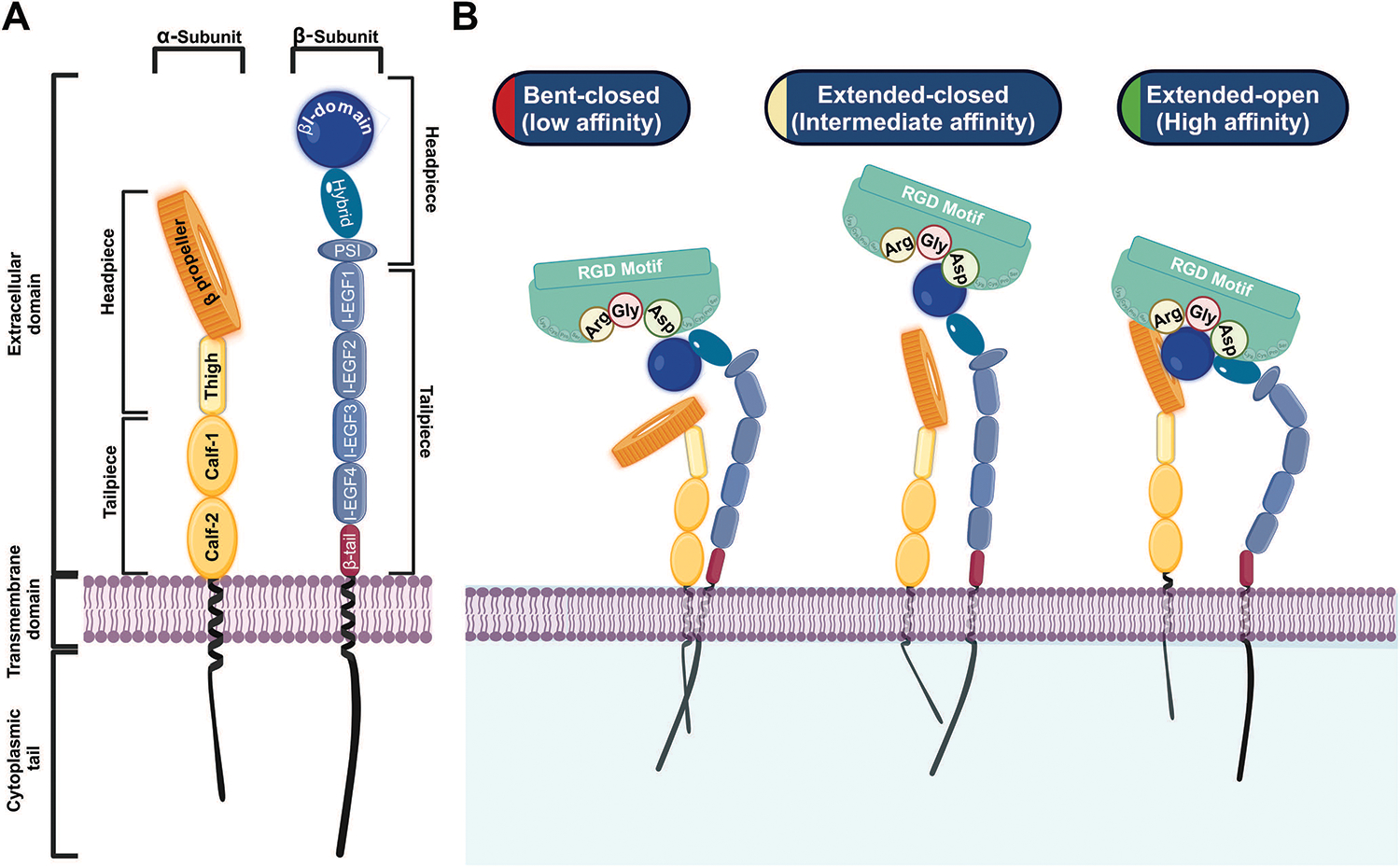
Structure and conformational states of RGD-binding integrins. **A:** Structural illustration of the RGD-binding integrin α-subunit (left, yellow) and β-subunit (right, blue). **B:** Conformational states showing transitions from bent closed (low affinity) to extended-open (high affinity), with the ligand-binding pocket interacting with the RGD motif. Biorender.com

Structurally, integrins are type I transmembrane proteins comprising three primary regions: a large extracellular domain, a single-pass transmembrane domain, and a short cytoplasmic tail (CT) (Fig. 1A) [10]. The extracellular domain of both the α and β subunits is divided into two parts: a headpiece and a tailpiece [11]. The α subunit headpiece features a seven-bladed β-propeller structure and a thigh domain, while its tailpiece contains the Calf-1 and Calf-2 domains [12]. The β subunit headpiece contains an inserted (βI) domain embedded within a hybrid domain and a plexin-semaphorin-integrin (PSI) domain. The tail of the β subunit consists of four cysteine-rich epidermal growth factor (EGF) modules and a β-tail domain (βTD) [11]. Ligand recognition and binding in RGD-binding integrins occur at a pocket formed by the interface between the β-propeller of the α subunit and the βI domain of the β subunit [11–13].

**Figure 2:**
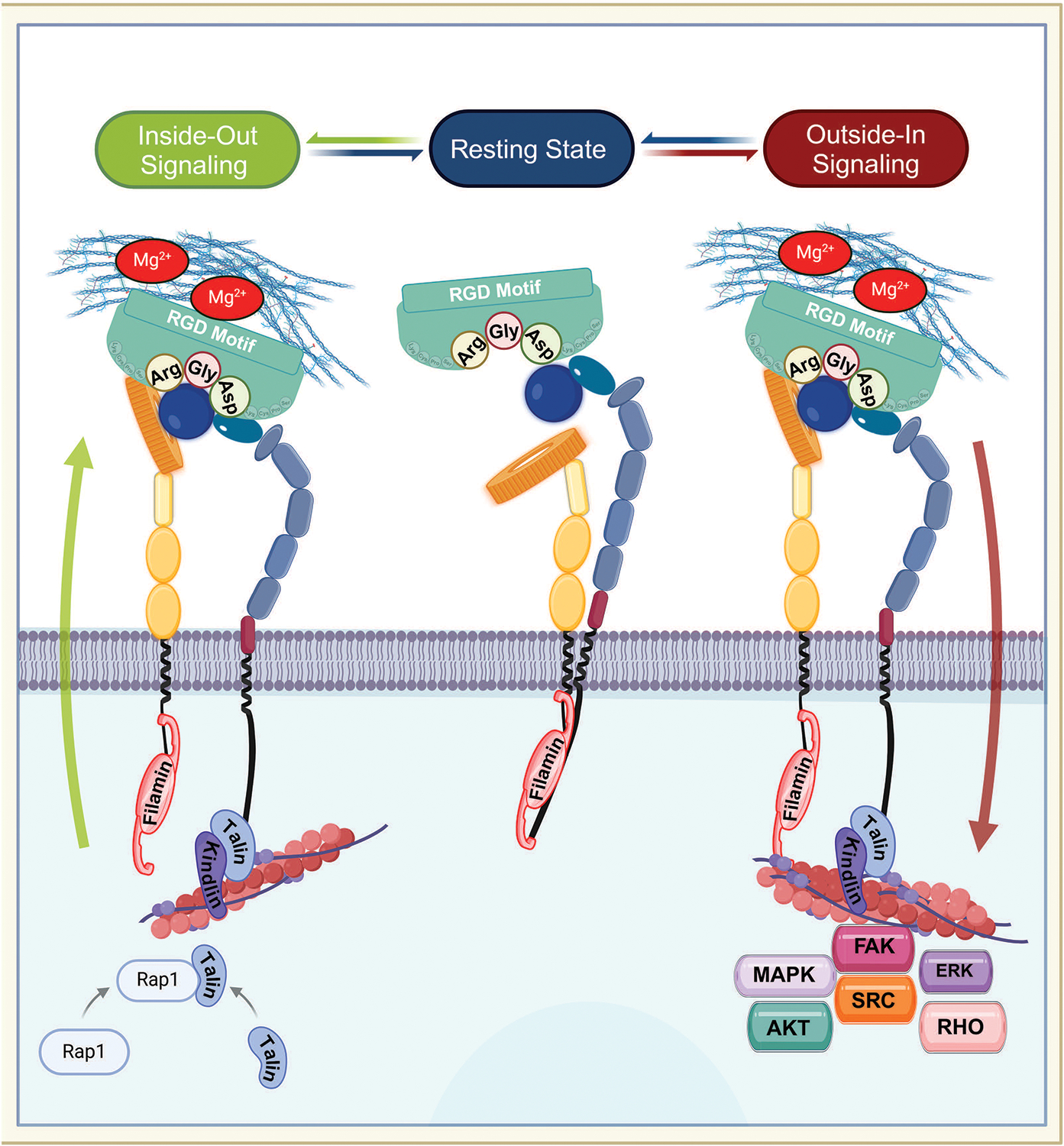
Bidirectional signaling of RGD-binding integrins. The figure illustrates the dynamic transitions of RGD-binding integrins through their resting, inside-out, and outside-in signaling states. In the resting state, integrins adopt a bent-closed conformation with low affinity, stabilized by filamin binding to both α and β subunits’ cytoplasmic tail (CT). Inside-out signaling begins when talin and kindlin bind the β-subunit CT, displacing filamin and inducing a conformational change to an extended-open state (high binding affinity). This process is enhanced by extracellular Mg^2+^ and mechanical ECM forces. In neutrophils, chemokine-activated Rap1 recruits talin to drive integrin activation. Upon ligand binding, integrins cluster at adhesion sites and initiate outside-in signaling, where filamin repositions to the a-subunit CT, linking integrins to actin filaments. This activates focal adhesion complexes and downstream pathways, including FAK, SRC, AKT, MAPK, ERK, and RHO, regulating adhesion, migration and cytoskeletal reorganization. Arg: arginine, Gly: glycine, Asp: asparagine. Created with Biorender

The maturation and heterodimerization of RGD-binding integrins, such as α8β1, occur in the endoplasmic reticulum, after which they are transported to the cell surface to carry out their functions [10,14–16]. RGD-binding integrins undergo dynamic conformational changes that facilitate their transition between different affinity states, which are crucial for their roles in adhesion and signaling. These states include the bent-closed (low-affinity) state, the extended-closed (intermediate-affinity) state, and the extended-open (high-affinity) state [17,18]. In the bent-closed state, the RGD-binding integrins adopt a compact structure with the extracellular domains folded close to the plasma membrane, resulting in low ligand-binding affinity [18]. This conformation is maintained in the absence of activating signals, ensuring that RGD-binding integrins remain inactive until required. The transition to the active extended-open state involves the complete opening of the headpiece, exposing the β-propeller of the α subunit and the βI domain of the β subunit [17,18]. This conformational shift significantly increases integrin’s affinity for the RGD-ligand containing motif, enabling stable adhesion and the activation of signaling cascades (Fig. 1B) [7,13,19,20]. A key feature of integrins is their ability to transduce signals bidirectionally between cells and their environment, linking extracellular cues to intracellular protein kinase activities [13]. RGD-binding integrins participate in two types of signaling: “inside-out signaling,” where they adjust the cell’s adhesive properties in response to internal signals, and “outside-in signaling,” where they regulate cellular processes in response to external cues (Fig. 2) [21]. Inside-out signaling is initiated by intracellular adaptor proteins such as talin and kindlin, which bind to the CT of the β-subunit. This binding destabilizes the interaction between the α and the β subunits [20–22], resulting in conformational changes that transition integrins from a bent, closed state (low affinity) to an open, extended state (high daffinity). Extracellular Mg^2+^ ions and mechanical forces from the ECM further enhance ligand-receptor affinity [19,23–25].

While talin was previously considered the primary adaptor protein responsible for integrin activation [26], recent studies demonstrate that the transmission of tensile forces is via a ligand-integrin-talin-actin cytoskeleton complex, essential for inside-out signaling [18]. This mechanism imparts mechanosensitive properties to integrins, exposing their binding sites under tensile conditions, which is critical for integrin activation during cellular adhesion and migration [27,28]. For example, in neutrophils, chemokine binding to G-protein-coupled receptors (GPCRs) activates Ras-related protein 1 (Rap1), a small GTPase that recruits talin and kindlin to the β-subunit’s CT, driving integrin activation [27,29]. Similarly, chemokine or T-cell receptor (TCR) stimulation triggers talin binding, enabling high-affinity interactions with ECM components. This fine-tuning of integrin activity is crucial for immune cell migration and chemotaxis [29].

Beyond its role in inside-out signaling, talin also facilitates the formation of focal adhesions (FAs) and their linkage to actin filaments, thus initiating outside-in signaling [30]. While the β subunit’s CT has traditionally been the primary focus for integrin activation studies, emerging evidence suggests that the α subunit’s CT also plays a significant role. The α subunit’s CT is occupied by filamin, which stabilizes integrins in their inactive state [30]. During activation, talin competes with filamin by binding to the β subunit’s CT, displacing filamin and promoting integrin activation. Once activated, filamin rapidly reorganizes to strongly associate with the α subunit, linking it to actin filaments. This process drives cytoskeletal reorganization and ensures firm cell adhesion, completing the cycle of outside-in signaling [30].

During outside-in signaling, ECM ligands activate integrins through mechanical forces, leading to integrin clustering at adhesion sites [31]. This clustering creates a hub for adaptor and signaling proteins, which triggers downstream signaling cascades involving molecules such as focal adhesion kinase (FAK) [9,32], sarcoma family kinases (Src) [33], protein kinase B (Akt) [32,34], extracellular signal-regulated kinase (Erk) [35–37], mitogen-activated protein kinase (MAPK) [35–37], and Rho-GTPases (RHO) [38–40]. These pathways regulate critical cellular functions, including cell survival, shape, polarity, and migration [35,36]. Additionally, RGD-binding integrins interact with growth factors, further expanding their roles in cellular signaling [35].

Filamin, a key adaptor protein, plays a dual role in bidirectional signaling by stabilizing inactive integrin complexes and promoting outside-in signaling activation [30]. To date, approximately 180 signaling, structural, and adaptor molecules have been identified in association with integrins, including kinases, Src-homology 2 (SH2)- and Src-homology 3 (SH3)-related molecules, GTPases, and phospholipid mediators [33]. Functionally, integrins serve as biochemical sensors that respond to ECM properties, thereby facilitating cell adhesion, migration, and signaling. These processes are critical for development, tissue homeostasis, and disease regulation [41,42]. Integrins also serve as receptors for growth factors, hormones, and polyphenols, further highlighting their versatility [7,43].

The specific interaction between integrins and their ligands represents a major therapeutic target. Recently, *in silico* screening of the of the protein data bank suggested that the RGD-binding integrins have two distinct binding sites: “Site1”, the classical binding site for the RGD-containing ECM proteins, and “Site 2”, an allosteric binding site for growth factors and pro-inflammatory mediators. Site 2 is primarily activated during platelet aggregation, and binding at this site can induce integrin activation in an allosteric manner, independent of canonical signaling pathways [44]. The therapeutic potential of integrins is well-documented, with seven integrin-targeting drugs currently available and nearly 90 drugs in clinical trials [7,43,45,46]. One promising approach involves the use of the internalizing RGD (iRGD) sequence therapy, which targets the surface of tumor endothelial cells. This strategy has shown significant potential in enhancing drug delivery to tumors [47–50].

Integrin α8β1, a member of the RGD-binding integrins, was first identified in chick nerves in the 1990s, where its α8 subunit was shown to bind exclusively with β1 to form the highly specific α8β1 complex [51–53]. In humans, integrin α8 shares significant structural similarities with other integrin α subunits, such as α5, αv, and αIIb [54]. It is predominantly expressed in contractile cell types, including vascular smooth muscle cells, neuronal cells, and mesangial cells [55]. This receptor interacts with a variety of ECM proteins, including fibronectin, nephronectin, osteopontin, vitronectin, and tenascin-C, with the highest binding affinity reported for nephronectin [56,57]. Integrin α8β1 plays a crucial role in modulating transforming growth factor beta (TGF-β) signaling, along with other downstream pathways necessary for development and cellular homeostasis [58]. Dysregulation of α8β1 activity has been implicated in several diseases, including fibrosis, cancer, and kidney dysfunction.

Although the functional role of integrin α8β1 in organ development and homeostasis remains poorly understood compared to other integrins, it is essential for processes such as cell adhesion, migration, and signaling, which are fundamental to tissue morphogenesis and repair. Its interactions with the ECM are particularly relevant in the pathophysiology of fibrosis and cancer metastasis. A deeper understanding of integrin α8β1 could reveal novel therapeutic strategies for diseases such as kidney fibrosis, cancer, and other disorders characterized by abnormal cellular behavior. Additionally, its involvement in immune modulation and tissue regeneration makes it a promising target for research in regenerative medicine. In this review, we aim to explore how integrin α8β1 regulates development, maintains homeostasis, and contributes to disease pathogenesis through specific signaling pathways, with the hope of inspiring new avenues for research and potential therapeutic interventions.

## 2 Role of Integrin *α*8*β*1 Expression in Tissues and Organs

### 2.1 Kidney

The kidney is a central organ in which α8β1 integrin plays a crucial role during morphogenesis, particularly by facilitating the mesenchymal-to-epithelial transition (MET), a key process in the establishment of the kidney’s functional architecture. This role of α8β1 was initially identified by Müller et al. [59]. The authors observed that, in mice at embryonic day 11.5 (E11.5), α8β1 is localized within the cap mesenchyme, surrounding the ureteric bud (UB), and interacts with ECM proteins such as nephronectin and fibronectin, although its affinity to fibronectin is approximately 100-fold lower than that of nephronectin. The authors proposed that fibronectin serves as a modulator of α8β1 activity, thereby fine-tuning the final nephron number during kidney formation [59].

The UB secrets nephronectin, which acts as a ligand for α8β1, forming a complex that activates critical signaling pathways, including the glial cell-derived neurotrophic factor (Gdnf) pathway. This pathway is essential for UB growth, branching, and nephron formation [60,61]. As development progresses from embryonic days E12.5 to E13.5, the activation of the MAPK/ERK signaling pathway, in conjunction with α8β1, supports the maintenance of nephron progenitor cells and ensures the structural integrity of the developing kidney [35,36].

**Figure 3:**
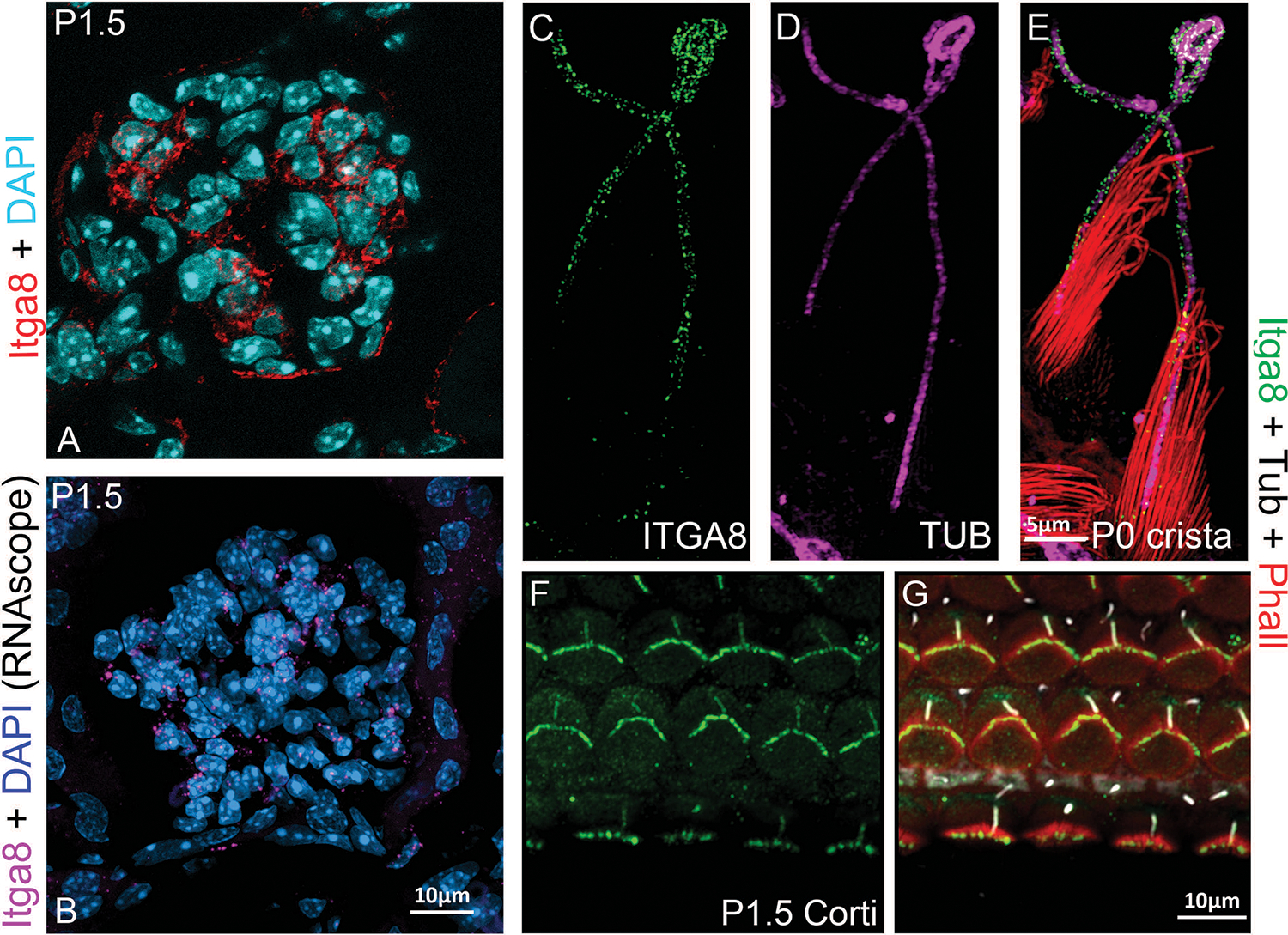
Integrin α8β1 expression and localization in the inner ear and kidney. **A–B**: α8β1 in the kidney localizes in the glomerular mesangial cells. A: Anti-α8 (red). B: RNAscope (Multiplex v2 Kit, Cat# 323271 ACDBio) for α8 (magenta). Sections were counterstained with DAPI. **C–E**: α8 in the vestibular system localizes at the cilia (magenta) and stereocilia (data not shown) levels. α8: green, phalloidin (Cat# A12381, ThermoFisher Sci.): red, and acetylated tubulin (Cat# T6793, Sigma-Aldrich): magenta. **F–G**: α8β1 in the organ of Corti, localizes in the hair cell bundle and cilia. α8β1: green, phalloidin: red and acetylated tubulin: white. Anti-α8 was a gift from Dr. L. Reichardt). Scale bars = **A–B**, **F–G**: 10 μm. **C–E**: 5 μm

Beyond its developmental roles, α8β1 continues to contribute to kidney homeostasis in adulthood. In the adult kidney, α8β1 is expressed in mesangial cells (Fig. 3A,B) within the glomerulus, where it plays a key role in maintaining homeostasis, facilitating phagocytosis, and promoting glomerular cell stability. Mesangial cells rely on α8β1 for cytokine production, debris clearance, and tissue repair processes [62,63]. Marek et al.. demonstrated that α8β1 enhances the phagocytic activity of mesangial cells, underscoring its importance in debris clearance and tissue healing [64–67]. Additionally, α8β1 modulates fibroblast activity and reduces immune cell infiltration during kidney injury through its effects on TGF-β levels and the activity of macrophage and T-cells [68].

Deficiency in α8β1 disrupts phagocytic capacity, likely due to alterations in cytoskeletal organization regulated by Rac1/rho-associated, coiled-coil-containing protein kinase 1 (ROCK1). This role in facilitating phagocytosis by renal mesangial cells is critical, and reduced α8β1 expression impairs phagocytosis and delays healing in mice [67]. This sex-specific phenotype suggests that hormonal factors may be influencing α8β1 activity. Notably, the absence of α8β1 in male mice results in smaller kidneys and reduced vascularization [69]. Studies using knockout animal models have further shown that loss of α8β1 leads to reduced phagocytic activity, delayed healing, and podocyte instability, highlighting integrin α8β1’s essential role in renal function from development through adulthood [66–68,70].

### 2.2 Lung

During lung embryogenesis, α8β1 integrin is highly expressed in the pleural basement membranes and the mesenchymal cells surrounding the branching airways, where it interacts with nephronectin and fibronectin [71,72]. These interactions are critical for airway branching and lobe separation during lung development [73–76]. Nephronectin, which strongly binds to α8β, has been shown to contribute to lung development by stabilizing ECM, maintaining airway branching, and supporting the structural distinction of lung lobes. In α8β1-deficient mouse models, these processes are disrupted, resulting in lung lobe fusion, abnormal collagen deposition, and pulmonary hypoplasia—a condition characterized by reduced lung size and inadequate airway branching [72,74].

In the adult lung, α8β1 expression is localized in stromal cells, fibroblasts, and alveolar basement membranes, where it plays a role in regulating tissue homeostasis, resolving inflammation, and supporting repair after injury [71,77]. The interaction of α8β1 with nephronectin facilitates ECM remodeling and promotes inflammation resolution during post-injury recovery [71,78]. In contrast, the absence of α8β1 impairs these processes, potentially leading to chronic inflammation and fibrosis, underscoring its importance in maintaining lung tissue integrity during repair [71].

### 2.3 Inner Ear

In the developing inner ear, α8β1 is expressed in the hair cells of both the vestibule (Fig. 3C–E) and the cochlea (Fig. 3F,G), localizing to their apical surface. α8β1 interacts with ECM components such as fibronectin to regulate and maintain the structural integrity stereocilia, the actin-filled protrusions that are essential for hair cell mechano-transduction, the process by which sound vibrations and head movements are converted into neural signals [79]. Global knockout of α8β1 disrupts fibronectin and FAK localization, both of which impair hair cell stability and function [9].

In zebrafish hair cells, α8β1 forms a complex with the stereo ciliary protein, protocadherin 15a (Pcdh15a), regulating cilia biogenesis and endocytosis via a RHOA-dependent mechanism [40]. Loss of α8β1 and Pcdh15a, either alone or in combination, leads to phenotypic defects such as ciliary elongation and impaired intracellular transport [40]. Genetic studies in humans have further highlighted the significance of α8β1 in auditory resilience, with the α8β1 variant rs10508489 linked to increased susceptibility to noise-induced hearing loss. This reinforces the importance of α8β1 activity in maintaining auditory function [80]. Additionally, α8β1 expression is upregulated during differentiation of human-induced pluripotent stem cells (hiPSCs), paralleling the expression of Sox2, a key transcription factor involved in hair cell differentiation [81]. This suggests that α8β1 may serve as a potential marker for hair cell development and maturation.

### 2.4 Liver

In liver development, α8β1 has been identified as a marker for a distinct population of hepatic stellate cells (HSC), as reported by Ogawa et al. In a murine model, the authors demonstrated that this HSC population plays a pivotal role in ECM remodeling and the progression of fibrosis [82]. Furthermore, α8β1 regulates lysyl oxidase-like 1 (LOXL1), a key enzyme involved in ECM stabilization and crosslinking. This regulation occurs through the activation of the FAK/PI3K/AKT/HIF1α signaling pathway, which promotes fibrosis under pathological conditions [32]. These findings highlight the involvement of α8β1 in the pathogenesis of liver fibrosis and suggest its potential as a therapeutic target in hepatic fibrotic diseases.

### 2.5 Other Tissues

#### 2.5.1 Brain

In the brain, α8β1 integrin plays a crucial role in neuronal development, particularly by regulating neurite outgrowth and hippocampal long-term potentiation (LTP), both of which are essential for neural network formation and cognitive function. During the development of chick embryos, α8β1 is highly expressed on the axon projections of immature sensory neurons, where it facilitates neurite outgrowth through its interaction with fibronectin [83]. Furthermore, genetic studies in humans have underscored the significance of α8β1 in neural health and disease. For instance, the α8β1 variant rs7077361 has been associated with reduced risks of Parkinson’s disease (PD), suggesting a potential neuroprotective role. Interestingly, α8β1 has also been linked to schizophrenia, though the findings exhibit variability across different populations and require further investigation to establish definitive correlations [84–86].

#### 2.5.2 Heart

In the heart, α8β1 is primarily expressed in interstitial fibroblasts and vascular smooth muscle cells (VSMCs), as well as in the epicardium, endocardium, and cardiac valves of rats, where it supports tissue structure and ECM stability under normal physiological conditions. Following angiotensin II (AngII) induction, α8β1 expression is significantly upregulated in myofibroblasts within the left ventricle and aorta. This upregulation promotes fibronectin and collagen deposition, contributing to ECM remodeling, tissue stiffness, and reparative fibrosis, which are critical for cardiac repair following injury or stress [87]. Interestingly, while α8β1 facilitates fibrosis through ECM regulation, its deletion does not entirely prevent fibrotic processes, suggesting that its role in cardiac function is complex and potentially influenced by compensatory mechanisms [87,88]. In adult VSMCs, α8β1 supports vascular adaptation to stress and maintenance of contractile function, which is essential for preserving blood vessel integrity and proper circulation [89].

#### 2.5.3 Lymphatic System

In the lymphatic system, α8β1 is vital for maintaining proper lymphatic contractility, which is crucial for preventing lymphatic dysfunction and associated complications. This contractility ensures effective lymph flow and overall fluid balance [90]. It plays a key role in maintaining vascular and lymphatic development by supporting contractility and adaptation to mechanical stress in vascular and lymphatic smooth muscle cells, as evidenced by genetic models demonstrating vascular dysfunction and aneurysm formation in the absence of α8β1 [89–91].

#### 2.5.4 Cornea

During corneal development, α8β1 regulates periocular neural crest (pNC) cell migration in chick embryos. In pNC cells, α8β1 binds to nephronectin through the RGD binding motif. This interaction facilitates pNC migration into the cornea via the FAK signaling pathway, a process that is essential for corneal formation. Experimental models demonstrated that blocking either FAK signaling or α8β1 activity resulted in impaired pNC migration, leading to significant corneal defects [92].

#### 2.5.5 Placenta

In the placenta, α8β1 contributes to vascularization and plays a regulatory role in placental development. During placenta-genesis, α8β1 is expressed in trophoblast cells, where it binds to fibronectin and osteopontin, participating in trophoblast migration. This is crucial for maintaining a functional placenta and ensuring healthy pregnancy outcomes [93].

#### 2.5.6 Intestine

During intestinal development, integrin α8β1 is expressed in proliferative epithelial cells located at the base of the crypts from 14 to 20 weeks of gestation. Through its binding to fibronectin, α8β1 promotes FAK integrity and stress fiber assembly via RHOA/ROCK pathway. This interaction enhances cell adhesion and proliferation while restraining cell migration, ensuring proper tissue organization and growth [94]. In the mature intestine, α8β1 expression becomes restricted to undifferentiated progenitor cells within the crypts, where it plays a key role in maintaining epithelial homeostasis [94]. This regulatory role extends beyond normal physiological conditions; for instance, during acute exposure to erythromycin, α8β1 is upregulated in intestinal epithelial cells as a compensatory mechanism to mitigate cytotoxicity and maintain tissue homeostasis [95].

#### 2.5.7 Dental Pulp

In dental pulp, α8β1 is highly expressed and regulates ECM formation and tissue adhesion, both of which are critical for maintaining the structural integrity of dental tissues [96].

#### 2.5.8 Overview

**Figure 4:**
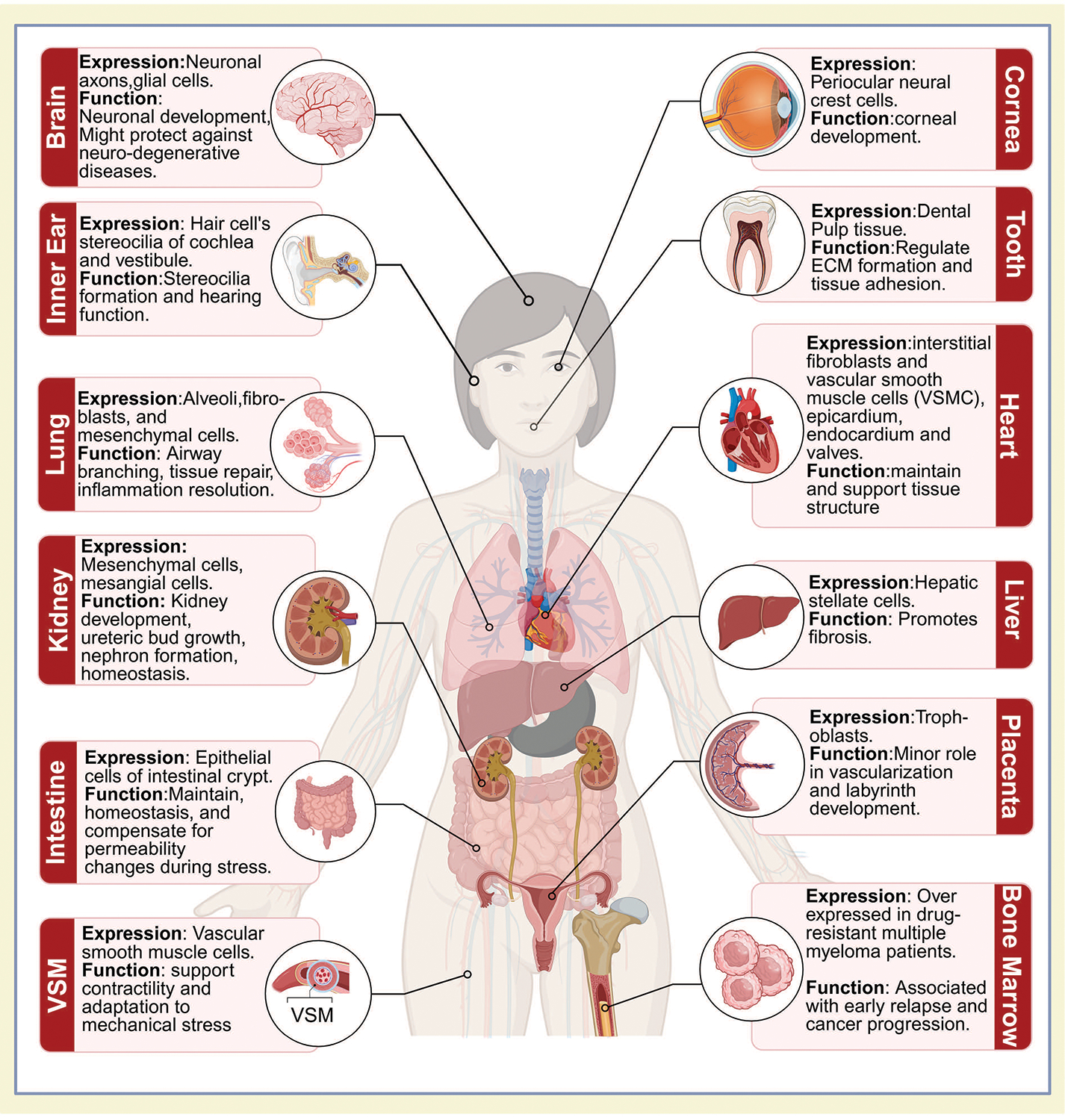
Illustration of integrin expression and function across organs: this figure represents α8β1 integrin expression and function across various organs. The anatomical diagram is based on human anatomy, with the expression data derived from both *in-vitro* and *in-vivo* studies. Created in https://BioRender.com

The anatomical diagram (Fig. 4) illustrates the expression pattern and function of α8β1 across various organs and tissues, based on data presented in Table 1. Altogether, integrin α8β1 plays diverse roles in tissue development, maintenance, and repair, contributing to processes such as MET in the kidney, ECM remodeling in the lung, neuronal development in the brain, and vascular adaptation in the heart. It is also involved in cell adhesion, migration, and tissue homeostasis in the placenta, cornea, dental pulp, intestine, and inner ear, where it regulates the stereocilia structure critical for mechano-transduction. While extensive research has elucidated the importance of α8β1 in the kidney during development, our understanding of its function in other organs, both during normal development and pathological conditions, remains limited. For example, while α8β1’s critical roles in lung tissue remodeling and inner ear function are well established, the underlying molecular mechanisms remain unclear. Despite these knowledge gaps, α8β1 shows great promise as a therapeutic target due to its diverse and tissue-specific roles during organ development and repair.

## 3 Role of Integrin α8β1 in Disease Pathogenesis in Tissues and Organs

Integrin α8β1 is recognized for its critical role in organ development and tissue homeostasis; however, its dysregulation is linked to a variety of pathological conditions, including congenital abnormalities, chronic fibrotic disease, degenerative disorders, and cancer. In the kidney, α8β1 deficiency in both humans and mice leads to impaired epithelial-mesenchymal interactions, resulting in severe congenital anomalies such as renal agenesis and hypoplasia, which significantly compromise renal function [97,100]. Structural mutations in the β-sheets Calf-1 and Calf-2 domains disrupt the receptor’s ability to interact with ECM components and have been associated with bilateral renal agenesis (BRA) in fetuses, often leading to miscarriage [97]. The effects of α8β1 mutations can persist into adulthood, with severity influenced by the location of the mutation within its 30 exons. Mutations in exon 28 and intron 13 of the α8β1 gene have been associated with severe congenital anomalies, such as end-stage kidney failure, as well as intellectual disabilities in humans [54,97,100]. However, less severe phenotypes are typically observed when mutations are limited to intron 13 [100].

In the liver, integrin α8β1 has been implicated in the progression of fibrosis in non-alcoholic fatty liver disease (NAFLD) through the activation of the RHOA signaling pathway. This pathway enhances ECM accumulation and stabilizes collagen by upregulating LOXL, with the FAK/PI3K/HIF1α signaling axis driving the activation of HSCs [32,103,106,107]. Dysregulated α8β1 expression in activated HSCs accelerates fibrosis and contributes to the progression of chronic liver disease [103,107]. Notably, the role of α8β1 in fibrosis is dynamic and can be modulated to attenuate disease progression. For example, miR-125b-5p has been shown to reduce fibrosis by downregulating LOXL1 and other pro-fibrotic markers [103,107]. Moreover, α8β1 contributes to liver fibrosis by enhancing the expression of Col1a1 and Col3a1 through RHOA signaling [103]. While α8β1 initially supports tissue repair, its sustained expression in fibroblast subtypes promotes pathological ECM remodeling and chronic fibrosis. The selective expression of α8β1 in activated HSCs makes it an attractive therapeutic target, as its modulation in diseased tissues reduces the risk of off-target effects, offering potential for precision medicine [108].

In lung adenocarcinoma (LUAD), α8β1 is often downregulated, correlating with poor prognosis and enhanced tumor progression [34,109]. This downregulation is thought to compromise ECM integrity and immune cell infiltration, thus creating a more permissive tumor microenvironment. Reduced α8β1 expression weakens the structural support of ECM and diminishes the infiltration of key immune cells, such as T cells and macrophages, which are crucial for effective anti-tumor responses. Restoring α8β1 expression could potentially inhibit tumor invasion and metastasis while improving immune cell infiltration. Moreover, α8β1 has been shown to interact with the phosphatidylinositol 3-Kinase (PI3K)/AKT pathway, influencing cellular proliferation and migration, further underscoring its importance in LUAD progression [34].

In idiopathic pulmonary fibrosis (IPF), α8β1 exhibits dynamic expression across different fibroblast subtypes. For example, CD24^+^/α8β1^−^ fibroblasts localize to collagen-rich connective regions, whereas CD48^−^/α8β1^+^ fibroblasts are found in elastin-rich regions. This spatial regulation suggests that α8β1 plays a specialized role in balancing collagen and elastin deposition, which are critical for lung ECM stability. The expression of α8β1 in these fibroblasts is positively regulated by TGF-β, a major driver of fibrosis [102]. Furthermore, a compensatory relationship between integrins α8β1 and α5β1 has been observed during IPF progression. In primary human lung fibroblasts (HLFs), silencing α5β1 significantly reduces cell proliferation and migration while simultaneously increasing α8β1 expression, particularly in older fibrotic tissue. This compensatory shift between α8 and α5 suggests a dynamic interplay in response to disease progression [110].

Regarding lung injury repair in chronic lung transplant rejection, α8β1 expression is notably low in the peri-bronchial region but highly expressed in the alveolar space, where it is thought to promote tissue repair and mitigate fibrosis [78]. In the inner ear, specific single nucleotide polymorphisms (SNP) in the α8β1 gene have been linked to an increased susceptibility to noise-induced hearing loss, such an example is the rs10508489 variant [80]. Moreover, α8β1 has been found to form a complex with Pcdh15a, a protein associated with syndromic and no syndromic hearing loss [40]. In vascular smooth muscle, α8β1 plays a key role in maintaining arterial and lymphatic integrity under normal physiological conditions, with its loss being associated with arterial pathologies such as abdominal aortic aneurysms (AAA) [89].

While α8β1 regulates and maintains tissue homeostasis under normal conditions, its dysregulation in cancer has led to its identification as both a prognostic marker and a potential therapeutic target for immunotherapy [111,112]. For instance, in human LUAD, the expression of α8β1 is significantly downregulated and correlates with poor prognosis due to a high mutation rate and epigenetic regulation [34], including methylation events. These modifications result in reduced α8β1 expression, contributing to shorter survival times, alterations in tumor microenvironment, and activation of key signaling pathways such as PI3k/AKT/mTOR, which promote tumor proliferation, invasion, and metastasis [34]. Furthermore, the interaction between α8β1 and FAK leads to ECM remodeling, facilitating tumor growth [113]. Beyond LUAD, the downregulation of α8β1 has been observed in several other cancers, including lung [109,114], breast [112], kidney [100], bladder [115], and colon cancer [105], and is generally indicative of poor prognosis [116]. Conversely, elevated α8β1 expression has been associated with enhanced immune infiltration and better response to immunotherapy. In LUAD, increased α8β1 levels correlate with improved prognosis, increased immune infiltration, and greater efficacy of immunotherapy [111] due to α8β1’s positive association with immune checkpoint genes and its role in facilitating immune cell infiltration [111]. CRISPR-Cas9 screening has identified α8β1 as a factor that sensitizes LUAD cells to abivertinib, a small molecule therapy that inhibits metastasis and improves therapeutic sensitivity [114].

In human’s multiple myeloma, high α8β1 expression has emerged as a novel prognostic marker, indicating early relapses and aggressive disease progression [104]. In this context, α8β1 upregulation is associated with early relapse and resistance to chemotherapeutic drugs such as melphalan and bortezomib [104]. In certa*in situ*ations, α8β1 can also contribute to disease progression. For example, in malignant mammary tumors in mice, both α8β1 and its ligand, tenascin-W, are upregulated during metastasis, facilitating the invasive spread of breast malignancies [116]. Similarly, studies have demonstrated that α8β1 expression is higher in adjacent normal tissue compared to tumor tissue, suggesting its potential as a diagnostic marker in colorectal adenocarcinoma (COAD) [105]. These varying patterns of α8β1 expression highlight the complexity of its role in cancer biology, with its expression serving as either a marker of aggressive disease or a therapeutic opportunity, depending on the cancer type and context.

To further investigate α8β1’s role, Warthi et al. have generated an α8β1-CreERT2 mouse line to achieve effective gene recombination across both sexes and targeted tissues. This model has proven to be an excellent tool for studying VSMC-specific gene functions, as it avoids complications such as VSMC-related pathologies seen in traditional knockout models [91]. Additionally, the α8β1-CreERT2 mouse line shows sex-independent activity, allowing for equal application in male and female mice [91]. Furthermore, it has shown the same specificity in targeting lymphatic smooth muscle genes [90]. These attributes make the α8β1-CreERT2 mouse model an invaluable tool for advancing research in vascular and lymphatic smooth muscle biology.

Overall, α8β1 plays a crucial role in regulating ECM interactions, cellular stability, and tissue repair. As a therapeutic target, α8β1 holds significant potential, with strategies such as microRNA-based modulation, pathway inhibitors, and gene therapy showing promise in mitigating disease progression. However, its complex and context-dependent roles require further investigation to fully understand its compensatory mechanisms and its involvement in pathological processes. In summary, α8β1 integrin is essential for maintaining organ development and tissue homeostasis, and its dysregulation underlies various pathological conditions, including congenital anomalies, fibrotic diseases, and cancer. In the kidney, α8β1 deficiency causes severe conditions like renal agenesis, hypoplasia, and end-stage kidney failure. In the liver, α8β1 promotes fibrosis in NAFLD through RHOA signaling and upregulation of fibrotic markers, while modulation via miR-125b-5p demonstrates its therapeutic potential. In LUAD, α8β1 downregulation correlates with poor prognosis, tumor progression, and reduced immune infiltration. Conversely, restoring its expression can inhibit metastasis and improve immunotherapy efficacy. Its dynamic expression in IPF underscores its role in fibroblast regulation and ECM balance, particularly through its interactions with α5β1 and TGF-β pathways. In the inner ear, mutations in α8β1 are linked to noise-induced hearing loss, and in vascular smooth muscle, α8β1 is essential for maintaining lymphatic integrity. In cancer, α8β1 exhibits dual roles, acting as a poor prognostic marker in cancers such as LUAD, whereas its high expression enhances immune infiltration and therapeutic outcomes. Research using the α8β1-CreERT2 mouse model has provided valuable insights into vascular roles, offering sex-independent specificity for targeted research. Despite its therapeutic promise, there remain gaps in understanding α8β1’s compensatory mechanisms, particularly in fibrotic diseases and its context-dependent roles in cancer.

## 4 Conclusion

Integrin α8β1 plays a pivotal role in organogenesis and tissue homeostasis, with its influence extending across multiple organs and developmental stages. Its critical importance is particularly evident in kidney development, where α8β1 facilitates ureteric bud growth, mesenchymal-to-epithelial transitions, and the cohesion of nephron progenitor cells—processes essential for the establishment of functional kidney architecture. Additionally, α8β1 contributes to the structural integrity of the kidney by modulating fibrosis and maintaining glomerular homeostasis. Beyond its role in the kidney, α8β1 is indispensable in lung development, where it regulates airway branching and lobe separation during embryogenesis. In adulthood, α8β1 continues to support tissue homeostasis, particularly in modulating inflammation resolution. In pathological conditions such as IPF and LUAD, α8β1 regulates fibroblast differentiation, ECM remodeling, and tumor progression, underscoring its potential as a therapeutic target. Its role extends to vascular integrity, where it preserves arterial and lymphatic stability, and to other less-studied tissues, such as the inner ear, the cornea, and the placenta, suggesting broader physiological relevance.

Despite these advances, several key knowledge gaps persist in understanding α8β1’s compensatory mechanisms, particularly in fibrotic diseases. Moreover, its context-dependent roles in cancer progression remain complex and poorly understood. Innovative models, such as the α8β1-CreERT2 mouse line, provide a valuable platform for precise gene targeting across tissues and sexes, offering new opportunities to study α8β1’s diverse functions. Further research into α8β1’s mechanisms could lead to targeted interventions that improve outcomes in fibrotic diseases, cancer, and other α8β1-related pathologies.

## Figures and Tables

**Table 1:** Role of integrin α8β1 in tissue and organs

Role of α8β1 in tissues and organs			
Organ	Expression and localization	Ligand	Main findings
**Kidney**	Mesenchymal cells of mouse from embryonic day E11-E16.5.	Fibronectin, vitronectin, tenascin-C	α8β1 supports ureteric bud growth; α8β1 knockout leads to nonfunctional kidneys [59].
	Metanephric mesenchyme of mouse, E11.5.	Nephronectin	α8β1 regulates GDNF activity and, thus, ureteric bud development; α8β1 knockout mice have a delay in bud invasion, leading to missing kidney [60].
	Cap mesenchyme around ureteric bud during kidney development.	Nephronectin	Human whole exome sequencing of in humans with bilateral renal agenesis showed mutations in α8β1 [97].
	Mouse fibroblast during kidney injury.	ND	α8β1 reduces fibroblast activation during kidney injury; α8β1 KO increases fibroblast activity, leading to sever kidney fibrosis [68].
	Mouse glomerular mesangial cells.	ND	Single-cell RNA sequencing of mouse glomerular mesangial cells revealed α8β1 as an extracellular matrix regulator [62,98].
	Mouse nephron progenitor cells (E12.5-E13.5).	Nephronectin	α8β1, through MAPK, helps kidney progenitor cells to properly interact with the surrounding tissue; α8β1 KO mice have defective kidneys [35].
	In mesenchyme of developing mouse kidney and mesangial cells in mature kidney.	Fibronectin, vitronectin, tenascin C, osteopontin, nephronectin	α8β1 maintains kidney homeostasis by supporting mesangial cell function and preventing cell death; α8β1 KO mice have smaller or missing kidneys [63].
	Podocytes and mesangial cells.	ND	α8β1 impacts glomerular morphology; α8β1 KO mice have impaired glomerular filtration [67].
	Mouse vascular smooth cells and kidney mesangial cells.	ND	α8β1 protects mouse blood vessels and kidney structure [99].
	Kidney metanephric mesenchyme.	ECM molecules with RGD motif	In humans, mutations in α8β1, cause renal hypodysplasia [100].
**Lung**	Mouse alveoli’s basement membrane.	Nephronectin	α8β1 regulates tissue homeostasis and repair during inflammation; α8β1 KO mice led to chronic lung inflammation and fibrosis [71].
	Alveolar space.	ND	α8β1 is highly expressed in the adult alveolar space [77].
	Mesenchymal cells surrounding branching airways (E15-E21).	ND	α8β1 is important for normal airway branching, and contribute to pulmonary hypoplasia, as shown in the α8β1 KO rat model [72,74].
	Stromal cells fibroblast, and pericytes.	ECM molecules with RGD motif: latent TGF-β peptide	α8β1 KO mouse models showed an increase in collagen deposition [101].
	Elastic fiber-rich connective tissue.	TGF-β signaling	α8β1 has a role in tissue remodeling and IPF by differentiating fibroblast subtypes in coordination with TGF-β signaling [102].
	Visceral pleura and mesothelial basement membrane (E12-early postnatally).	Nephronectin	α8β1 signaling is responsible for lung lobe separation during embryonic development; α8β1 KO mice have lobe fusions and abnormal collagen deposition [72].
**Inner ear**	Mouse cochlea and vestibular hair cells (E16 to post-natal day 3)	Fibronectin, tenascin C, osteopontin	α8β1 regulates stereocilia formation, fibronectin localization, and FAK signaling [9].
	Zebrafish hair cell.	ND	α8β1 colocalizes with Pcdh15a to regulate cilia biogenesis through a RHOA-dependent mechanism [40].
**Liver**	In hepatic stellate cells (HSCs) found in fibrotic regions during liver fibrosis.	ND	α8β1 KO has a decrease in HSC activation and ECM remodeling, leading to a delay in fibrosis progression [82].
	Upregulated HSC during fibrosis.	ND	Through FAK/PI3k/AKT/HIF1α pathway, α8β1 modulates LOXL1, promoting ECM stabilization and liver fibrosis progression [32].
	Upregulated in NAFLD patients and mouse model.	ND	Through RHOA signaling, α8β1 promotes liver fibrosis in NAFLD; miR-125b-5p has been suggested as a therapeutic agent to inhibit liver fibrosis by reducing α8β1 levels [103].
**Brain**	α8β1 is expressed in neurons and glial cells during development.	ND	An association between α8 rs7077361 variant and reduced risk of Parkinson’s disease [85].
**Heart**	Expressed in the interstitial fibroblasts and vascular smooth muscle cells of the normal rodent myocardium.	Fibronectin	Upon angiotensin II-induced fibrosis upregulates α8β1 expression in myofibroblasts, enhancing ECM production and promoting fibrosis. While α8β1 plays a key role in matrix deposition and cardiac remodeling, its deletion does not fully prevent fibrosis [87].
**Cornea**	Periocular neural crest cells during development.	Nephronectin	α8β1 is essential for corneal development by regulating neural crest migration [92].
**Bone marrow**	Upregulated in early-relapsed multiple myeloma patients.	ND	α8β1 KO have a reduction in migration, invasion, and drug resistance in cancer cells [104].
**Colon**	Upregulated in normal tissue surrounding colon adenocarcinoma patients.	ND	α8β1 acts as a prognostic marker for colon adenocarcinoma [105].
**Intestine**	Upregulated in intestinal epithelial cells when exposed to a high dose of erythromycin.	ECM component	α8β1 upregulation acts as a protective modulator for epithelial permeability once exposed to erythromycin [95].
	Expressed in human proliferative epithelial cells of the crypt between gestational weeks 14–20; restricted to undifferentiated progenitor cells in the mature intestine.	Fibronectin	α8β1 maintains intestinal epithelial homeostasis by promoting adhesion proliferation and migration regulation via RHOA/ROCK signaling. While its knockdown disrupts homeostasis and impairs intestinal function [94].
**Placenta**	Expressed in placental throphoblasts of humans, mice, and rats.	Fibronectin, osteopontin	α8β1 plays a minor role in placental development and vascularization [93].
**Tooth**	Expressed in human dental pulp tissue.	ND	Increased α8β1 in dental pulp compared to periodontal ligament. Linked to ECM and adhesion pathways to support tissue repair [96].

Note: IPF: idiopathic pulmonary fibrosis, NAFLD: non-alcoholic fatty liver disease, ND: not determined.

## Data Availability

Data sharing does not apply to this article as no datasets were generated or analyzed during the current study.
